# Slip Boundary-Enabled Multiscale Modeling for Sound Absorption Coefficient of Nanofiber Porous Media with High Fidelity

**DOI:** 10.3390/nano15221696

**Published:** 2025-11-09

**Authors:** Jiangming Jin, Bohan Cao, Jietao Huang, Liyang Jiang, Ziyi Liu, Tairong Kuang, Wei Wu, Feng Chen, Yanpei Fei

**Affiliations:** 1 College of Mechanical Engineering, Zhejiang University of Technology, Hangzhou 310014, China; jjm@zjut.edu.cn (J.J.); altoriacbh@163.com (B.C.); 201906040411@zjut.edu.cn (L.J.); 2 Zhejiang Key Laboratory of Advanced Polymer Materials Modification and Application Technology, College of Material Science and Engineering, Zhejiang University of Technology, Hangzhou 310014, China; huangjietao200311@163.com (J.H.); l1847907261@163.com (Z.L.); kuangtr@zjut.edu.cn (T.K.); chenf@zjut.edu.cn (F.C.); 3 ZHANBOQIANYAN New Material Technology Co., Ltd.^®^, Jiaxing 314205, China; tybb6667@yahoo.co.jp; 4 Zhejiang Joysun Advanced Material Co., Ltd., Jiaxing 314205, China

**Keywords:** nanofibers, sound absorption coefficient, multiscale finite element simulation, slip boundary condition

## Abstract

Nanofibers, with their lightweight structure and superior sound absorption, are promising materials for noise control in automotive and architectural applications. However, due to the complex porous structure of nanofibers, established acoustic models often fail to accurately quantify the microstructure’s influence on sound absorption characteristics, resulting in substantial prediction errors. To determine the sound absorption characteristics of nanofibers, an equivalent fiber network model was developed using the multiscale finite element analysis (MFEA) method based on SEM images of nanofibers. The slip boundary condition (SBC) was then applied to calculate the microstructural parameters necessary for macroscopic characterization. The sound absorption coefficients of nanofibers were characterized using three acoustic models, and the results were compared with the experimental data. The predictions of the Limp frame model agreed well with the experimental data within the 500–6400 Hz frequency range. Through use of the multiscale model developed in this study, a deterministic relationship between microstructure and acoustic properties was established, revealing that the inertial interactions between sound waves and the nanofiber skeleton, as well as the slip boundary effect at the nanofiber surfaces, are among the primary mechanisms contributing to the flow resistance and superior sound absorption performance of nanofibers.

## 1. Introduction

Fibrous materials, as broadband sound absorbers, are widely used in noise control applications, such as in the automotive industry (e.g., headliners and door panel absorbers) and as architectural acoustic barriers. However, conventional fibrous materials have significant limitations in terms of low-frequency noise control. The primary challenge in this regard is that achieving superior sound absorption coefficients at low frequencies requires the material’s thickness to exceed one-quarter of the incident wavelength [1]. This requirement results in increased volume and weight, which conflicts with the spatial constraints in automotive manufacturing and architectural designs. As a result, there is an urgent need to develop lighter, thinner fibrous materials that exhibit superior sound absorption performance in low-frequency bands.

The microstructural design of nanofibers offers a novel approach to resolving the aforementioned issues. The nanoscale pore structure of nanofibers imparts elevated flow resistance, which significantly enhances acoustic energy dissipation, thereby enabling superior sound absorption performance in low-frequency bands, which is difficult for conventional fibrous materials to attain. Therefore, compared to conventional fibrous materials, nanofiber porous media exhibit greater potential in practical noise control applications. In the automotive field, nanofiber layers—such as polypropylene (PP) or polyvinylidene fluoride (PVDF) nanofibers—can be applied in engine covers, door panels, and ceiling structures to reduce interior noise while maintaining lightweight design. In architectural acoustics, nanofiber composites can be used in wall panels and interior finishing systems to enhance broadband sound absorption performance while maintaining low structural thickness. There are various nanofiber fabrication processes, such as blow spinning, electrospinning, self-assembly, template methods and phase separation [2]. Among the spinning techniques, blow spinning is achieved through high-speed airflow stretching of the polymer solution, where shear forces induce bending instability in the polymer jet. Simultaneously, solvent evaporation occurs within the precursor system, ultimately leading to the solidification and deposition of micro-nanofibers onto the collector [3]. Unlike electrospinning, blow spinning enables the fabrication of nanofibers with thinner and more uniform diameters at room temperature; as there is no requirement for the use of a high-voltage electric field, this method enhances safety and broadens the scope of nanofiber applications.

To gain deeper insight into the acoustic performance of nanofibers, many researchers have focused on advancing the development of acoustic characterization methods tailored to these materials. Horoshenkov et al. [4] employed flow resistivity as the input parameter to establish a lumped element model for thin nanofiber layers, revealing a marked disparity between experimentally measured flow resistivity and that derived from acoustic characterization. Akasaka et al. [5] employed dedicated testing devices to determine the microstructural parameters of nanofibers and combined the Johnson–Champoux–Allard (JCA) model with Biot’s theory to predict sound absorption coefficients, where good agreement with the experimental results was exhibited. Building on Horoshenkov’s work, Hurrell et al. [6] employed the Biot and Darcy models to characterize thin nanofiber layers on porous substrates. Their measurements revealed significant discrepancies between experimentally determined flow resistivity and model predictions, demonstrating that the classic Kozeny–Carman flow resistivity model is no longer applicable to nanofibers. Ulrich et al. [7] further corroborated this perspective through performing an experimental investigation of nanofiber layers adhered to porous substrates. Chen et al. [8] employed the JCA and lumped element models to characterize nanofiber–porous composite structures, revealing significant discrepancies between experimentally measured flow resistance and that obtained through acoustic characterization.

The aforementioned studies indicate that conventional acoustic models for porous materials exhibit significant errors when characterizing the acoustic properties of nanofibers, with there being two different reasons for these limitations: Firstly, macroscopic characterization methods fail to fully account for the influence of microstructure on the sound absorption characteristics of nanofibers due to their unique physical properties (e.g., sorption effects [9]), arising from nanoscale diameters. Secondly, the determination of microstructural parameters necessitates extensive datasets and specialized ultrasonic instrumentation [10], resulting in substantial costs and susceptibility to boundary condition interference. These challenges suggest that to improve the prediction accuracy of nanofiber acoustic performance, it is essential to develop numerical approaches that can directly link nanoscale flow behavior and microstructural features to macroscopic acoustic performance. To address these issues, microstructural imaging techniques [11] have emerged as an effective tool with which to characterize fiber microstructures. These techniques establish critical connections between microscopic fiber architectures and macroscopic acoustic characteristics, providing innovative pathways for nanofiber research. For instance, He et al. [12] systematically investigated the influence of fiber diameter, length, orientation, and porosity on the sound absorption performance of glass wool by reconstructing an equivalent fibrous network. Du et al. [13] combined multiscale finite element modeling with scanning electron microscopy imaging to study the effects of pressure-induced-flow processing on the sound insulation properties of polypropylene/polyolefin elastomer composite foams. Their research revealed that the multilayered impedance mis-match structure significantly improved sound transmission loss. Multiscale modeling, as demonstrated in their study, provides an effective bridge between microstructural morphology and macroscopic acoustic functionality. Previous works have indicated that there exists an optimal scale range where the correlation between material structure and functionality is strongest [14]. Building upon these findings, the present work employs a multiscale finite element analysis framework to identify the critical scales linking nanofiber microstructure to sound absorption behavior.

Despite considerable advances in modeling fibrous materials, conventional multiscale models still have limitations in describing nanofiber porous media. Owing to nanoscale pore sizes and interfacial slip-flow phenomena, these models often lead to large deviations in estimating internal flow-field distributions, thereby affecting the accuracy of key microstructural parameters required for acoustic prediction [15,16].

In this study, a three-dimensional microstructure model of nanofibers was developed based on scanning electron microscopy (SEM) images, and a multiscale finite element analysis (MFEA) framework incorporating the slip boundary condition (SBC) was established to accurately represent nanoscale flow characteristics and compute microstructural parameters. These parameters were subsequently transferred into an macroscopic acoustic model to calculate the normal-incidence sound absorption coefficients. Compared to conventional models, the proposed approach (1) quantitatively links SEM-derived microstructural features with acoustic parameters to improve the prediction accuracy of nanofiber sound absorption coefficients, and (2) provides physical insights into nanoscale flow mechanisms that influence nanofiber acoustic energy dissipation. The significance of this work lies in its bridging of microscopic fiber morphology and macroscopic acoustic functionality, offering a predictive design tool for lightweight, high-performance acoustic materials in automotive and architectural noise-control applications.

## 2. Experimental Section

### 2.1. Materials

**Figure 1:**
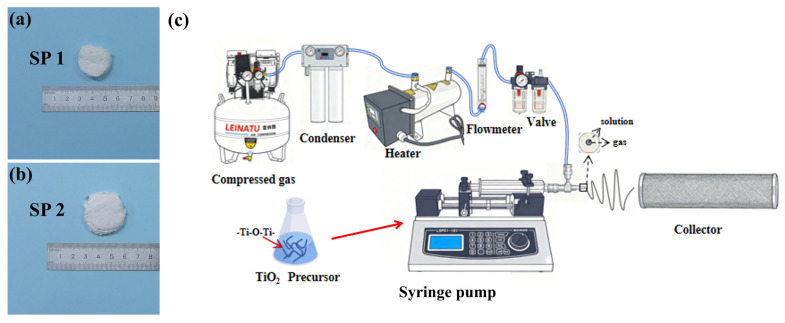
(**a**) Commercial polypropylene nanofiber; (**b**) laboratory-synthesized TiO_2_ nanofiber; (**c**) schematic of the blow spinning method.

In this study, a comparative analysis of two nanofiber specimens was performed: commercial polypropylene nanofibers (SP 1) supplied by ZHANBOQIANYAN (Zhejiang, China) New Material Technology Co., Ltd.^®^ and laboratory-synthesized TiO_2_ nanofibers (SP 2); this is shown in Figure 1a,b.

The TiO_2_ nanofibers were fabricated using a blow spinning system, as illustrated in Figure 1c. The precursor solution was prepared by dissolving polyvinylpyrrolidone (PVP, Mw ≈ 1,300,000, Macklin, Shanghai, China) in anhydrous ethanol (≥99.7%, Sinopharm Chemical Reagent Co., Ltd., Shanghai, China) and glacial acetic acid (AR, Hushi Chemical Co., Ltd., Shanghai, China) with a solvent mass ratio of 2:1 to 5:1. The PVP concentration was maintained between 3 and 10 wt%. After full dissolution and clarification, tetrabutyl titanate (Ti(OBu)_4_, ≥99%, Aladdin, Shanghai, China) was gradually added as the titanium source, and the mixture was stirred for 2 h until a homogeneous, transparent yellow sol was obtained.

The precursor solution was then transferred into a coaxial nozzle equipped with a 28G inner needle and injected using a multi-channel syringe pump (LONGER, Baoding, China) at a flow rate of 1.0–5.0 mL/h. Compressed air supplied by a Leinatu HS50L-1100W air compressor (0.8 MPa, 204 L/min) was used to drive airflow at 20–25 m/s, with ambient relative humidity controlled at 30–50%. The airflow was monitored using a glass-type flowmeter (0.16–1.6 m^3^/h), and heated to maintain stable jet formation via an air heater (220 V, 2.7 kg). The tip-to-collector distance was maintained at 20 cm to ensure complete fiber drying and uniform deposition during blow spinning.

The as-spun TiO_2_ nanofiber mats were collected on an aluminum-foil-covered rotating drum (diameter 100 mm, rotation speed 500 rpm) and dried in a convection oven at 80 °C for 12 h to remove residual solvents. The dried samples were subsequently calcined in a tube furnace (GSL-1400X-S, Hefei Kejing Materials Tech Co., Ltd., Hefei, China) under air or nitrogen atmosphere. The temperature was increased at a rate of 1–5 °C/min in a stepwise heating profile and maintained at 500–600 °C for 120–220 min. After calcination, the furnace was allowed to cool naturally to room temperature.

Both the SP 1 and SP 2 samples were cut into 29 mm diameter circular disks using a precision round cutter to fit the impedance tube cross-section for acoustic testing. The bulk density (*ρ*) of each specimen was measured using a JHY-S300 solid–liquid densitometer. Because the nanofibers dissolve in water, a sealed test cell was employed to prevent direct contact with the liquid. The densitometer automatically calculated the density by measuring the weight of the sealed box before and after loading the nanofibers, and the difference represents the bulk density of the nanofiber.

To verify the accuracy of the geometric calculation, the measured porosity (*ϕ_m_*) was determined according to the Chinese National Standard GB/T 42697—2023 [17] as

(1) $$\phi_{m}=1-\frac{\rho }{\rho_{fiber}}$$

where *ρ* is the bulk density of the specimen and *ρ*_fiber_ is the density of the nanofibers. The measured bulk density and porosity of the samples are summarized in Table 1.

### 2.2. Measuring Equipment

**Figure 2:**
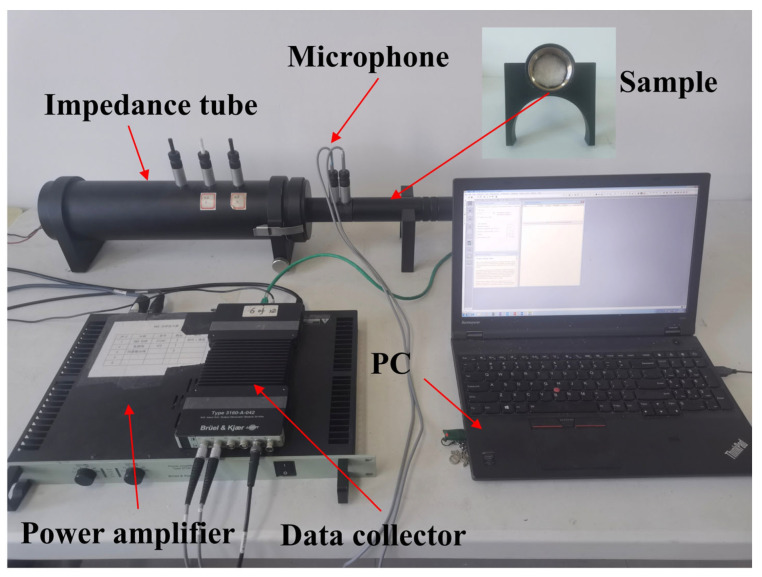
Two-microphone impedance tube for absorption coefficient measurement.

The setup for measuring the nanofiber sound absorption coefficient is shown in Figure 2. For acoustic testing, a Brüel & Kjær (B&K) Type 4206 two-microphone impedance tube system (Brüel & Kjær, Virum, Denmark) was employed. A random noise signal was generated via a 3560 signal generator (Brüel & Kjær, Virum, Denmark), amplified through a Type 2716C power amplifier (Brüel & Kjær, Virum, Denmark), and measured using two Type 4187 microphones (Brüel & Kjær, Virum, Denmark) positioned at specific axial distances (the distance from the sample surface to the nearest microphone is 70 mm) within the impedance tube. Each sample was placed flush against the rigid termination at the downstream end of the impedance tube to ensure normal incidence. The samples were mounted using a silicone O-ring to guarantee airtight sealing and prevent leakage around the edges. Each measurement was repeated five times, and the average value was used for analysis. The ambient temperature and relative humidity during testing were 23 ± 1 °C and 50 ± 3%, respectively.

The sound absorption coefficient was calculated in compliance with the ISO 10534-2 standard [18]. A small acoustic tube with an inner diameter of 29 mm was employed to ensure plane wave propagation, enabling measurements across the 500–6400 Hz frequency range. Detailed equipment specifications and experimental procedures are provided in the Supplementary Materials (Figure S1 and Table S1).

## 3. Theory and Methods

### 3.1. Characterization and Modeling

**Figure 3:**
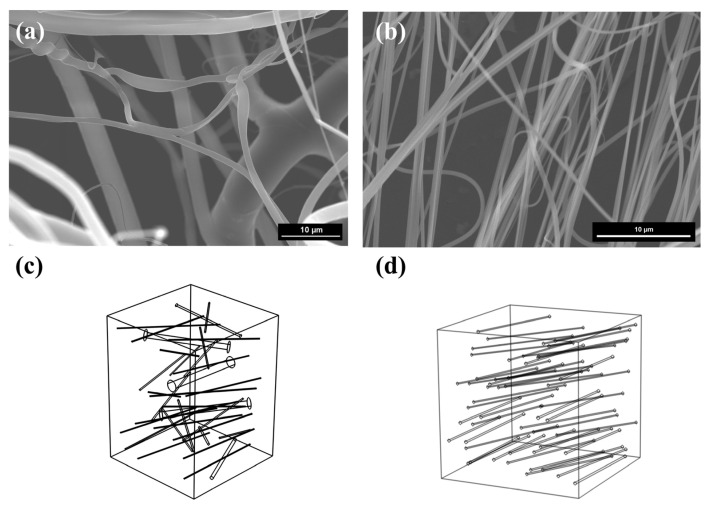
(**a**) SEM image of commercial polypropylene nanofibers (SP 1); (**b**) SEM image of blow spinning nanofibers (SP 2); (**c**) 3D microstructure model of commercial polypropylene nanofibers (SP 1); (**d**) 3D microstructure model of blow spinning nanofibers (SP 2).

Scanning electron microscopy (SEM) images of commercial polypropylene nanofibers (SP 1) and TiO_2_ nanofibers (SP 2) are shown in Figure 3a,b.

Quantitative statistical analyses of the parameters—including fiber diameter, quantity, length, and spatial orientation—were conducted using ImageJ image-processing software (Version 1.54f).

Prior to parameter extraction, each SEM image was converted to 8-bit grayscale and segmented through adaptive thresholding to separate the fiber phase from the background. A median filter was then applied to reduce image noise and eliminate isolated particles. The processed images were binarized and skeletonized to extract individual fiber segments, from which the fiber length and number density were calculated. The fiber diameter was measured by manually selecting fibers per sample, and Gaussian distributions were fitted to the measured data to determine the mean and standard deviation. Fiber orientation was analyzed using the OrientationJ plugin in ImageJ to obtain the angular distribution of fiber axes relative to the reference direction. The results showed that the average fiber diameter of SP 1 was approximately 946 nm, whereas SP 2 exhibited a significantly finer average diameter of 310 nm, along with enhanced uniformity compared to SP 1.

In order to develop a multiscale prediction of the acoustic characteristics of nanofibers, accurate calculations of microstructural parameters such as flow resistivity (*σ*), tortuosity (*α_∞_*), viscous characteristic length (Λ), and thermal characteristic length (Λ’) are required. In this study, a MATLAB R2019b-based nanofiber microstructure modeling program was developed within a numerical-homogenization framework. The measured fiber diameter distribution, fiber length, and 3D orientation angles were first extracted from SEM images and converted into probability density functions, which were then used as statistical inputs for the generator. Within a fixed-size cubic grid space representing the reconstructed 3D fiber model, fibers were simplified as straight cylindrical rods, ignoring the influence of fiber curvature on fluid flow behavior. For each fiber, the program (i) randomly samples a fiber center inside the cube, (ii) randomly samples an orientation vector from the SEM-based orientation probability density functions, and (iii) assigns a fiber length within the experimentally observed range together with a diameter drawn from the measured distribution. The fiber axis is then constructed along the sampled orientation; if part of the fiber extends outside the cube, only the segment lying inside the computational domain is kept. A simple overlap/collision check is applied to avoid nonphysical intersections with previously placed fibers. The procedure is repeated until both the porosity and the number of fibers fall within a prescribed tolerance of the experimental values. The geometric characteristics of individual fibers were uniquely defined by their diameter, central coordinates (X_C_, Y_C_, Z_C_), and three-dimensional orientation vector **v**. The final microstructure model is shown in Figure 3c,d.

### 3.2. MFEA Model

This study adopts a hierarchical multiscale approach that connects microstructural geometry to macroscopic acoustic properties through parameter transfer. At the microscopic scale, scanning electron microscopy (SEM) images provide the fiber geometry and pore morphology used for numerical reconstruction. At the mesoscopic scale, multiscale finite element analysis (MFEA) is employed to compute the flow resistivity from a Stokes-flow problem with a slip boundary condition, while tortuosity and the viscous characteristic length are obtained from a potential-flow problem that is independent of viscous boundary effects. These parameters are then incorporated into macroscopic acoustic models (D–B–M, JCA, and Limp Frame Model) to predict sound absorption behavior.

The 3D microstructure model of the nanofibers was imported into COMSOL Multiphysics 6.2^®^, and airflow in the normal direction was simulated by solving the Stokes equations to evaluate flow resistivity [19]. In addition, a potential (inviscid) flow field was separately derived within the same geometry to determine the tortuosity (*α_∞_*) and viscous characteristic length (Λ).

The characterization of the nanofiber fluid boundary slip effect is a critical aspect of this study. While previous research has proposed various microscale fluid flow prediction methods [20,21], their applicability to nanofibers remains limited: conventional models typically adopt the no-slip boundary condition (NSBC), neglecting the velocity slip effect at the fiber–fluid interface. This leads to underestimated fluid velocity predictions, thereby compromising the accuracy of microstructural parameters such as flow resistivity. Various studies have shown that when the gas mean free path (λ) becomes comparable to the fiber diameter (*d_f_*), the continuum assumption is no longer valid, meaning that velocity slip corrections need to be introduced [22]. Recent experimental investigations have confirmed the presence of slip effects in nanofiber media through pressure-dependent flow measurements, further validating the necessity of the slip boundary condition in computational models [23]. The Stokes equations in the void space among the fibers under slip boundary condition (SBC) are described as follows:

(2) $$\begin{array}{l} -\mu \Delta u+\nabla p=0\;(\mathrm{momentum}\;\mathrm{balance})\\ \nabla \cdot u=0\;(\mathrm{mass}\;\mathrm{conservation})\\ n\cdot u=0\;\mathrm{on}\;\Gamma \;(\mathrm{no}\;\mathrm{flow}\;\mathrm{into}\;\mathrm{fibers})\\ t\cdot u=-L_{s}n\cdot \nabla (u\cdot t)\;\mathrm{on}\;\Gamma \;(\mathrm{slip}\;\mathrm{flow}\;\mathrm{along}\;\mathrm{fibers})\\ P_{in}=P_{out}+c\;(\mathrm{pressure}\;\mathrm{drop}\;\mathrm{is}\;\mathrm{given}) \end{array}$$

where ***n*** is the normal direction to the fiber axis, ***u*** represents the periodic velocity, *L*_s_ represents the slip length, *μ* is the dynamic viscosity of air, *P*_in_ and *P*_out_ represent the inlet and outlet pressures of the nanofiber, *c* denotes the constant pressure drop imposed across the computational domain, and ***t*** denotes an arbitrary tangential direction satisfying ***t***⋅***n*** = 0.

To quantitatively evaluate the significance of rarefied gas effects in nanofibers, the Knudsen number (Kn) was calculated for both specimens:

(3) $$Kn=\frac{2\lambda }{d_{f}}$$

where *λ* is the gas mean free path, taken as 38.5 nm for air at standard temperature and pressure based on recent molecular dynamics results [24,25], and *d_f_* is the fiber diameter. Using this value, the Knudsen numbers are calculated as 0.08 for SP 1 and 0.25 for SP 2. Both values fall within the slip flow regime (0.001 < Kn < 0.3), indicating that rarefied gas effects, including velocity slip, remain significant. However, SP 2 lies close to the upper bound of this range and may exhibit partial transition effects, which could affect the accuracy of flow resistivity calculations in the subsequent MFEA model.

In summary, since the fiber diameter of the nanofibrous material is comparable to the gas mean free path, rarefaction effects become significant, requiring the use of slip boundary conditions in the microscopic flow analysis. In this study, the flow field was therefore obtained by solving the Stokes equations in COMSOL Multiphysics 6.2^®^, with SBC introduced at the fiber-fluid interface to characterize nanoscale fluid flow. The slip velocity (*U*_w_) on the fiber surface was calculated using a first-order Maxwell model, as follows:

(4) $$U_{w}=\left(\frac{2-\sigma_{v}}{\sigma_{v}}\right)\lambda \frac{\partial u}{\partial n}$$

where *σ*_v_ is the momentum accommodation coefficient.

**Figure 4:**
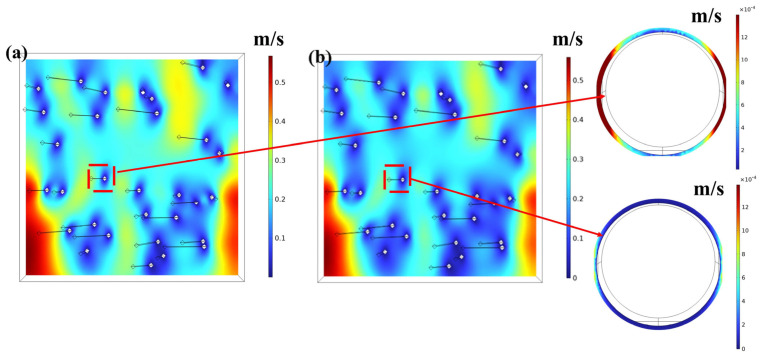
Flow velocity in nanofiber media under different boundary conditions: (**a**) slip boundary condition (SBC); (**b**) no-slip boundary condition (NSBC). The color map represents the magnitude of local fluid velocity.

A comparison of velocity field distributions under slip boundary condition (SBC) and no-slip boundary condition (NSBC) is shown in Figure 4. Under the NSBC, the fluid velocity relative to the fiber surface is strictly zero (no-slip condition), resulting in steep velocity gradients near the fibers. In contrast, the SBC allows for a finite tangential velocity slip at the fiber surfaces.

Assuming a homogeneous nanofiber microstructure, the four boundaries along the flow direction were set as symmetric boundary conditions. A constant pressure difference of 25 Pa was applied to the inlet and outlet boundaries. The flow resistivity (*σ*) was calculated as follows:

(5) $$\sigma =\frac{\Delta p}{Vh}$$

where Δ*p* denotes the pressure drop between the inlet and outlet, *h* is the sample thickness, and *V* is the depth-averaged steady flow velocity derived from the Stokes equations under an applied pressure gradient. To provide a comparative verification, the flow resistivity under the no-slip boundary condition (NSBC) was also calculated under otherwise identical conditions.

The porosity (*ϕ*) was calculated by determining the pore volume of the nanofibers:

(6) $$\phi =\frac{V_{p}}{V_{t}}$$

where *V_p_* is the volume occupied by the fluid (air), and *V_t_* is the total volume of the domain under consideration. In COMSOL Multiphysics 6.2^®^, the pore volume (*V_p_*) were obtained by subdomain corresponding to the air phase using the Integration Component Coupling operator. The total volume (*V_t_*) was calculated directly from the geometric dimensions of the computational domain.

Tortuosity (*α_∞_*) is a dimensionless parameter that characterizes the complexity of the pore space. It is an inherent characteristic of the porous skeleton [26].

The tortuosity was calculated according to the definition proposed by Johnson et al. [27], which is based on the potential flow solution within the pore space:

(7) $$\alpha_{\infty }=\frac{\langle (\nabla \phi )∙(\nabla \phi )\rangle }{{\left|\langle \nabla \phi \rangle \right|}^{2}}$$

where ∇*ϕ* is the gradient of the velocity potential defining the potential flow field, *v_m_ =* ⟨∇*ϕ*⟩ represents the macroscopic velocity, and ⟨⋅⟩ represents the volume average operator, which denotes the spatial averaging of a field variable over the Representative Volume Element (RVE).

The viscous characteristic length (Λ) and thermal characteristic length (Λ’) are key parameters characterizing the viscous dissipation and thermal dissipation properties of porous media. The viscous characteristic length quantifies the dissipation of acoustic energy through viscous losses within the pores, while the thermal characteristic length characterizes the thermal exchange efficiency between the pore skeleton surface and the fluid. The calculation formulas for the viscous and thermal characteristic lengths are as follows:

(8) $$\begin{array}{l} \Lambda =2\frac{\int_{V_{p}}{\left|\nabla \phi \right|}^{2}dV}{\int_{S_{p}}{\left|\nabla \phi \right|}^{2}dS}\\ \Lambda^{\prime }=2\frac{V_{p}}{S_{p}} \end{array}$$

where *V_p_* is the pore volume and *S_p_* is the effective surface area of the pores.

**Figure 5:**
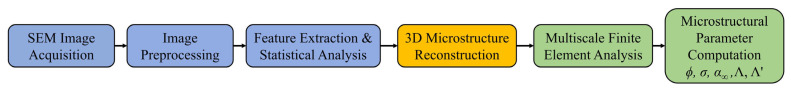
Workflow of SEM image acquisition to microstructural parameter calculation.

Before proceeding, the limitations of the MFEA model should be noted. The current model assumes idealized cylindrical fibers without considering curvature or surface roughness, therefore slightly underestimating local flow resistance. In addition, the Representative Volume Element (RVE) used in the simulation is limited in size due to computational cost, and may not fully capture the long-range connectivity and orientation distribution of fibers in real samples. Moreover, potential sorption effects between the fiber surfaces and air molecules are not yet included in the present model, which may slightly influence the acoustic prediction. Furthermore, fiber bundling could locally alter pore connectivity and flow channels, potentially introducing deviations in the calculated microstructural parameters. These simplifications could lead to deviations when extrapolating microscopic parameters to macroscopic acoustic behavior. A schematic workflow illustrating the process from SEM image acquisition to microstructural parameter calculation is provided in Figure 5 for clarity.

### 3.3. Acoustic Characterization of Nanofibers

To characterize the acoustic properties of nanofibers, this study employs three acoustic prediction models for porous materials: the Delany–Bazley–MiKi (D–B–M) model, the Johnson–Champoux–Allard (JCA) model, and the Limp Frame Model.

The normal incidence sound absorption coefficient (α) of the material is expressed as follows:

(9) $$\alpha =1-{\left|\frac{Z_{N}-\rho_{f}c_{0}}{Z_{N}+\rho_{f}c_{0}}\right|}^{2}$$

where *ρ_f_* is the air density, c_0_ is the speed of sound of air, and *Z_N_* is the normal acoustic impedance of the material, given by the following:

(10) $$Z_{N}=-jZ_{c}\cot(kh)$$

where *h* is the material thickness, *Z*_c_ is the characteristic impedance, and *k* is the complex wave number.

The characteristic impedance (*Z*_c_) and complex wave number (*k*) are determined via the effective density (ρ(ω)) and bulk modulus (*K*(ω)) of the porous material’s effective fluid model:

(11) $$Z_{c}=\sqrt{\rho (\omega )K(\omega )}$$

(12) $$k=\omega \sqrt{\frac{\rho (\omega )}{K(\omega )}}$$

Delany and Bazley proposed an empirical model based on flow resistivity (Delany–Bazley Model, D–B) [28] to calculate the characteristic impedance and complex wave number of porous media. Miki further revised this model by adjusting the exponential coefficients of the frequency-dependent terms, addressing the issue of potential negative real parts of acoustic impedance in the original model. The improved model is referred to as the Delany–Bazley–Miki (D–B–M) model [29] and it employs the following formulation for the approximation of acoustic characteristics:

(13) $$Z_{c}=\rho_{f}c_{0}\left[1+5.501{\left(\frac{f}{\sigma }\right)}^{-0.612}-j8.431{\left(\frac{f}{\sigma }\right)}^{-0.612}\right]$$

(14) $$k=\frac{\omega }{c_{0}}\left[1+7.811{\left(\frac{f}{\sigma }\right)}^{-0.618}-j11.41{\left(\frac{f}{\sigma }\right)}^{-0.618}\right]$$

where ω is the angular frequency, *ρ*_f_ is the air density, c_0_ is the speed of sound of air, *f* is the frequency, and *σ* is the flow resistivity.

The Johnson–Champoux–Allard (JCA) model [30] is a rigid-frame model, which assumes that the fibrous medium is more rigid than the fluid medium. Consequently, the porous medium can be treated as an equivalent fluid flowing within a rigid frame. This model is based on five acoustic parameters: porosity (*ϕ*), flow resistivity (*σ*), tortuosity (α_∞_), viscous characteristic length (Λ), and thermal characteristic length (Λ′). The effective density (ρ(ω)) and bulk modulus (*K*(ω)) in the JCA model are expressed as follows:

(15) $$\rho (\omega )=\frac{\alpha_{\infty }\rho_{f}}{\phi }\left[1+\frac{\sigma \phi }{i\omega \rho_{f}\alpha_{\infty }}\sqrt{1+\frac{4i\omega \alpha_{\infty }^{2}\mu \rho_{f}}{\sigma^{2}\Lambda^{2}\phi^{2}}}\right]$$

(16) $$K(\omega )=\frac{\gamma p_{A}}{\phi }{\left(\gamma -(\gamma -1){\left[1+\frac{8\mu }{i\omega \Lambda \prime^{2}\mathrm{Pr}\rho_{f}}\sqrt{1+\frac{i\omega \Lambda \prime^{2}\mathrm{Pr}\rho_{f}}{16\mu }}\right]}^{-1}\right)}^{-1}$$

where Pr is the Prandtl number, *γ* is the ratio of specific heats of air, *ρ*_f_ is the air density, *p*_A_ is the standard atmospheric pressure, and *μ* is the dynamic viscosity of air.

The Limp Frame Model [31], proposed by R. Panneton as an extension of the JCA model, explicitly incorporates the inertial effects of the solid phase in flexible porous media. By eliminating the rigid-frame assumption while neglecting the elastic stiffness of the skeleton, this model characterizes the mass coupling between the fluid and a movable solid phase that responds inertially to acoustic excitation without elastic restoring forces. This formulation significantly enhances accuracy in predicting the sound absorption properties of flexible materials (e.g., nanofibers) through improved representation of inertial interactions.

The Limp Frame Model eliminates the rigid skeleton assumption required by the JCA model but introduces six parameters from Biot’s theory. The effective density and bulk modulus of the Limp Frame Model are calculated as follows:

(17) $$\rho (\omega )=\frac{\rho^{\prime }(\omega )\rho_{t}-{\rho_{f}}^{2}}{\rho_{t}+\rho^{\prime }(\omega )-2\rho_{f}}$$

(18) $$\rho \prime (\omega )=\frac{\alpha_{\infty }\rho_{f}}{\phi }\left[1+\frac{\sigma \phi }{i\omega \rho_{f}\alpha_{\infty }}\sqrt{1+\frac{4i\omega \alpha_{\infty }^{2}\mu \rho_{f}}{\sigma^{2}\Lambda^{2}\phi^{2}}}\right]$$

(19) $$\rho_{t}=\rho +\phi \rho_{f}$$

(20) $$K(\omega )=\frac{\gamma p_{A}}{\phi }{\left(\gamma -(\gamma -1){\left[1+\frac{8\mu }{i\omega \Lambda \prime^{2}\mathrm{Pr}\rho_{f}}\sqrt{1+\frac{i\omega \Lambda \prime^{2}\mathrm{Pr}\rho_{f}}{16\mu }}\right]}^{-1}\right)}^{-1}$$

where *ρ*_t_ is the effective mass of the equivalent fluid and *ρ* is the bulk density of the material, calculated as follows:

(21) $$\rho =\frac{m}{h}$$

where *m* is the surface density of the material and *h* is the sample thickness.

The main symbols and parameters used in Section 3 are summarized in Abbreviation.

## 4. Results and Discussion

### 4.1. Microstructural Parameter Analysis

The microstructural parameters used for the acoustic characterization of nanofibers are summarized in Table 2, and they were obtained through multiscale finite element analysis based on 3D microstructural models reconstructed from SEM-derived geometric parameters.

**Figure 6:**
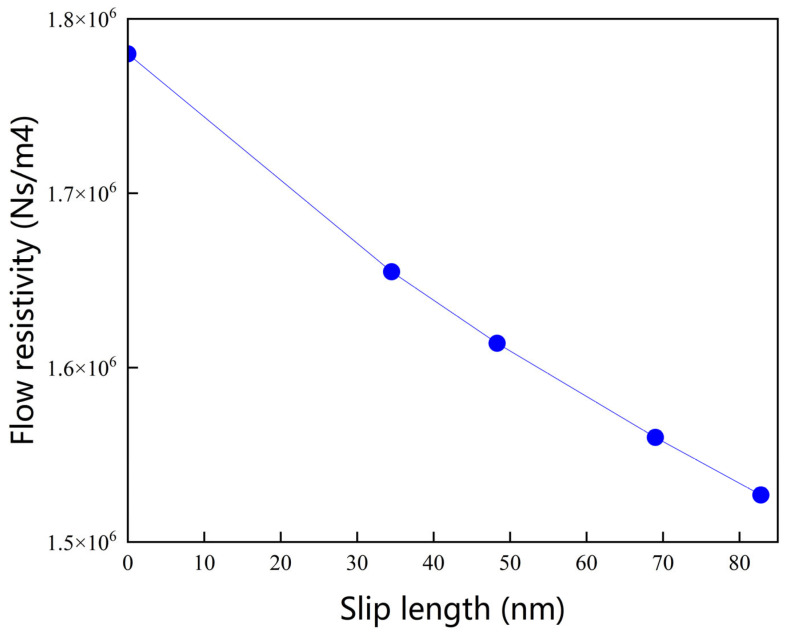
Relationship between slip length and flow resistivity for nanofiber structures.

The implementation of the SBC critically influences the key microstructural parameters governing acoustic performance. Comparative analysis reveals a reduction in *σ* for both nanofiber samples under the SBC relative to the NSBC; this is attributed to the underprediction of pore gas flow velocities in nanofibrous media using NSBC models. To better illustrate the effect of slip flow on macroscopic acoustic performance, the relationship between slip length and flow resistivity is shown in Figure 6. The slip length characterizes the degree of velocity slip at the fiber surface, with zero slip length corresponding to the classical no-slip condition. Under the slip boundary condition, the variation in flow resistivity directly reflects the influence of slip flow on the acoustic dissipation behavior of nanofiber materials. The slip boundary condition (SBC) allows gas molecules to move more easily near the fiber surfaces, thereby facilitating deeper airflow penetration into the complex pore network of the nanofiber layer. This effect is reflected in the flow resistivity behavior: although nanofibers maintain significantly higher flow resistivity than conventional fibrous materials (typically 10^3^–10^5^ Ns/m^4^), the overall flow resistivity decreases gradually as the slip effect becomes more pronounced. Therefore, the incorporation of slip boundary condition is essential for the accurate prediction of the sound absorption performance of nanofiber materials.

In practice, the direct measurement of microstructural parameters such as tortuosity (*α_∞_*), and characteristic lengths (Λ, Λ′) requires specialized ultrasonic instruments. Therefore, the microstructural parameters in this study were compared with previously reported literature data [5,32] to validate the former’s reliability. The obtained values fall within the typical ranges for nanofibrous porous materials, confirming the physical soundness of the adopted calculation method. Nonetheless, the use of calculated rather than directly measured parameters may introduce minor uncertainties in the subsequent acoustic predictions. To further clarify this validation, a comparative summary of the computed parameters and representative literature values has been added as Table 3.

Overall, the microstructural parameters used in this study were derived from SEM-based geometric data and subsequently computed through the multiscale finite element framework, thereby establishing a direct and physically grounded link between the nanoscale morphology and the macroscopic acoustic properties.

### 4.2. Prediction and Experimental Comparison

**Figure 7:**
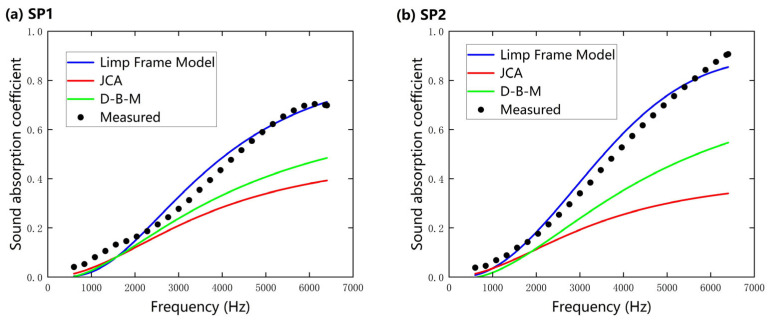
A comparison of the normal incidence sound absorption coefficients of nanofibers predicted using the Delany–Bazley–Miki (D–B–M), Johnson–Champoux–Allard (JCA), and Limp Frame Model under slip boundary condition (SBC) and experimental test data (3 mm). (**a**) SP 1; (**b**) SP 2.

Based on the microstructure parameters of nanofibers obtained via multiscale calculation under the slip boundary condition (SBC), a comparison between the sound absorption coefficients determined using the three acoustic prediction models and the experimental data for 3 mm thick samples is shown in Figure 7. The selection of these three models is justified by the differences in their theoretical foundations: the D–B–M model relies solely on flow resistivity, which simplifies computation but neglects other microstructural parameters; the JCA model enhances prediction accuracy by introducing physical quantities such as porosity and tortuosity at the expense of increased computational complexity; and the Limp Frame Model further incorporates fiber skeleton vibrations.

It can be observed that the results predicted by the Limp Frame Model align closely with the experimental results, whereas the JCA and D–B–M models exhibit significant deviations in the high-frequency range (>2000 Hz). This indicates that the airflow within the fiber pores is influenced not only by viscous friction but also by inertial coupling with the fibrous skeleton due to the lighter weight, lower stiffness, and higher flow resistivity of the nanofibers. Therefore, as it incorporates the limp-frame effective density, the Limp Frame Model is more suitable for characterizing the acoustic properties of thin nanofibers compared to conventional rigid-frame models. Furthermore, the consistent trends between the predictions of all three models and the experimental data validate the accuracy of the predictions, demonstrating that the multiscale prediction method proposed in this study achieves a balance between computational efficiency and prediction accuracy.

Moreover, a direct comparison between the two nanofiber specimens revealed that SP 2 exhibited higher overall sound absorption coefficients than SP 1. This enhancement can be attributed to the former’s finer fiber diameter and higher porosity, which lead to greater flow resistivity and intensified viscous and thermal dissipation. The SEM observations indicate that SP 2 has a more compact and uniform morphology, promoting stronger viscous dissipation and enhanced heat exchange in the confined pores. Therefore, reducing fiber diameter within a nanofiber network can effectively enhance the sound absorption capability of nanofibers.

**Figure 8:**
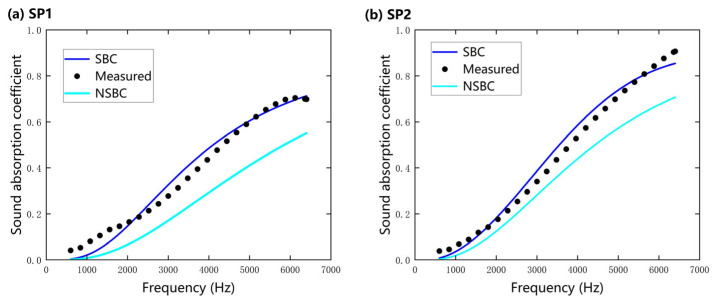
A comparison of experimental normal-incidence sound absorption coefficients of nanofibers and those predicted by the Limp Frame Model under slip and no-slip boundary conditions (3 mm): (**a**) SP 1; (**b**) SP 2.

The essential role played by slip boundary condition (SBC) is detailed in Figure 8, which shows the experimental normal-incidence sound absorption coefficients and those predicted by the Limp Frame Model under both slip and no-slip boundary conditions for 3 mm thick samples. For both nanofiber specimens, predictions under no-slip boundary condition (NSBC) consistently underestimate the absorption coefficient, with the discrepancy being most pronounced in the high-frequency range (>2000 Hz). In contrast, SBC-based predictions closely follow the experimental results across the entire 500–6400 Hz spectrum. This improvement arises because the SBC provides a more realistic description of fiber–fluid interactions; in particular, SBC achieves the following:

(1) It captures slip velocities near nanofiber surfaces, which increase interfacial friction between air molecules and fibers and thereby enhance acoustic energy dissipation;

(2) It better reproduces the tortuous propagation paths of sound waves through the nanofiber network;

(3) It yields microstructural parameters that more faithfully reflect nanofiber morphology.

In summary, these effects demonstrate that incorporating slip-flow mechanisms is indispensable for the accurate acoustic characterization and prediction of nanofiber materials.

**Figure 9:**
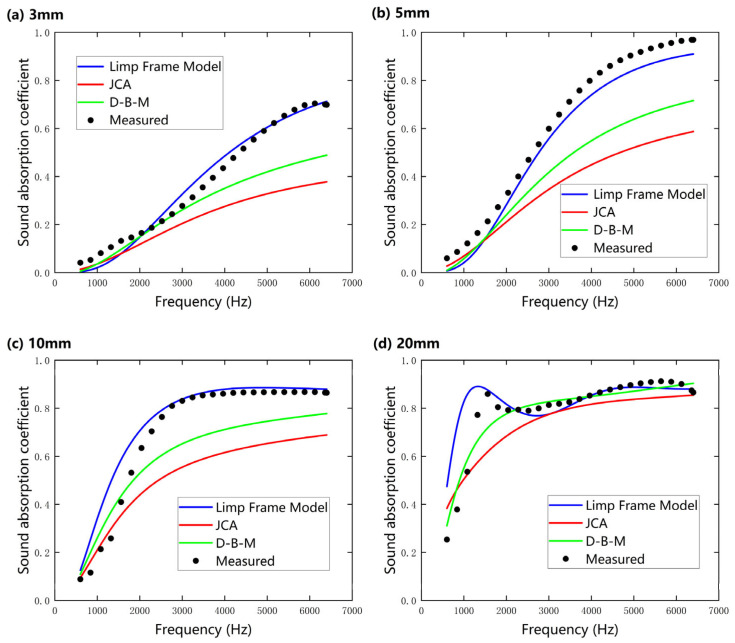
A comparison of the normal-incidence sound absorption coefficients of nanofibers predicted using the Delany–Bazley–Miki (D–B–M), Johnson–Champoux–Allard (JCA), and Limp Frame Models under slip boundary condition, compared with experimental measurements at different sample thicknesses (SP 1): (**a**) 3 mm; (**b**) 5 mm; (**c**) 10 mm; (**d**) 20 mm.

Figure 9a–d present a comparative analysis of the sound absorption coefficients obtained through acoustic model predictions and experimental measurements for nanofibers under the slip boundary condition (SBC) at sample thicknesses of (a) 3 mm, (b) 5 mm, (c) 10 mm, and (d) 20 mm. The results reveal that as the sample thickness increases from 3 mm to 20 mm, the average sound absorption coefficient within the 500–6400 Hz range significantly improves, which is attributed to the enhanced viscous and thermal conductive dissipation processes between air and fibers. Notably, 20 mm nanofibers exhibit an average sound absorption coefficient of 0.83, with a prominent absorption peak (sound absorption coefficient α ≈ 0.9) predicted by the Limp Frame Model in the low-frequency range (<2000 Hz), consistent with the experimental trend but slightly higher in magnitude. This result not only confirms the low-frequency sound absorption advantage of nanofibers over traditional fibrous materials under low-thickness conditions but also highlights the correlation between the absorption peak and their unique microstructure, where enhanced viscous–thermal dissipation and inertial coupling dominate acoustic energy dissipation mechanisms. In particular, the finer fiber diameter, higher porosity, and flow resistivity of the nanofiber samples contribute to increased inertial interactions between the oscillating air and the nanofiber skeleton, leading to broader effective absorption bands and higher absorption peaks.

Across the measured frequency range, the absorption behavior exhibits a clear transition between different dominant mechanisms. At low frequencies (<2000 Hz), sound absorption is primarily influenced by inertial effects caused by the interaction between fibers and air, leading to a distinct absorption peak. As frequency increases (2000–6400 Hz), viscous losses gradually become the major contributor, as the relative motion between air and fiber surfaces leads to continuous energy dissipation through shear interactions, resulting in broadband and high sound absorption. As reported in previous papers [33,34,35], it is thought that nanofibers can be vibrated by incident sound due to their low stiffness, which results in a distinct peak frequency of the sound absorption coefficient.

Furthermore, as the sample thickness increases (>10 mm), the prediction errors of the D–B–M and JCA models gradually decrease, with their overall curves approaching those of the Limp Frame Model. This phenomenon can be attributed to the increased framework density of the nanofibers and the reduced proportion of elastic medium within the pores, which causes the effective density *ρ*(ω) to converge toward *ρ*’(ω). Under these conditions, the contribution of skeleton vibrations to acoustic energy dissipation becomes negligible, with the dissipation predominantly governed by fluid viscous effects. However, the Limp Frame Model, which incorporates the inertial contribution of the solid phase, exhibits significantly lower prediction errors in the peak frequency bands compared to rigid-frame models like D–B–M and JCA, highlighting the critical role of characterizing microscopic vibration modes to enhance model accuracy.

**Figure 10:**
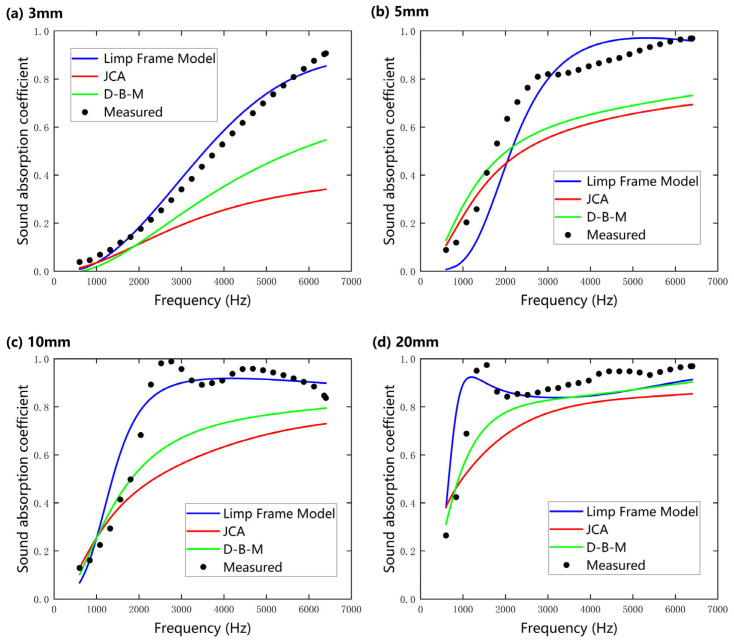
A comparison of the normal incidence sound absorption coefficients of nanofibers predicted using the Delany–Bazley–Miki (D–B–M), Johnson–Champoux–Allard (JCA), and Limp Frame Models under slip boundary condition, compared with experimental measurements at different sample thicknesses (SP 2): (**a**) 3 mm; (**b**) 5 mm; (**c**) 10 mm; and (**d**) 20 mm.

Similar simulations were performed for SP 2, with the corresponding results presented in Figure 10. Compared with SP 1, SP 2 exhibits systematically higher absorption over the measured band, and its primary absorption peak is slightly shifted toward lower frequencies. To quantify this difference, the arithmetic mean of the normal-incidence absorption coefficient ⟨α⟩ was computed over 500–6400 Hz (Table 4). The experimental results show that ⟨α⟩ of SP 2 exceeds that of SP 1 at all investigated thicknesses (3, 5, 10, and 20 mm), suggesting that the finer fiber diameter of SP 2 promotes stronger viscous and thermal energy dissipation across the spectrum.

When comparing model predictions, the Limp Frame Model reproduces both the peak position and magnitude more accurately for SP 2 than the rigid-frame models (JCA and D–B–M), consistent with the significant inertial coupling between the air and the fiber skeleton.

## 5. Conclusions

In this study, the relationship between nanofiber microstructure and macroscopic acoustic behavior was quantitatively analyzed using SEM-based reconstruction and multiscale finite element analysis (MFEA). The findings reveal that as the fiber diameter approaches the mean free path of air molecules, microscale slip flow becomes a key factor influencing acoustic energy dissipation. Incorporating the slip boundary condition (SBC) into the model enables accurate estimation of the critical microstructural parameter—flow resistivity—effectively bridging nanoscale flow physics with macroscopic sound absorption. Among the three tested models, the Limp Frame Model achieved the best agreement with experimental results, underscoring the crucial role of inertial coupling between the air phase and the compliant nanofiber skeleton in determining low-frequency sound absorption performance. Based on the comparison between the Limp Frame Model predictions and experimental results for nanofiber mats with different fiber diameters, the sound absorption coefficient increases with decreasing fiber diameter over the 500–6400 Hz band, indicating that finer fibers enhance viscous and thermal energy dissipation—a mechanism accurately captured by the Limp Frame Model. Moreover, nanofiber mats with sufficient thickness exhibit a pronounced absorption peak in the low-frequency region. The vibration of the fibers induced by incident sound waves is considered a possible mechanism responsible for this absorption peak.

This work establishes a quantitative framework linking nanoscale fiber geometry to macroscopic sound absorption performance and demonstrates that the proposed multiscale MFEA model can accurately predict the acoustic properties of nanofiber materials. The model not only provides design guidance for tailoring nanofiber structures with targeted acoustic characteristics but also reveals the influence of nanoscale flow slip on the acoustic dissipation behavior of nanofibers, thereby explaining the high flow resistivity and superior absorption characteristics observed in fine nanofiber networks. The findings of this study provide practical guidance for the structural design and performance optimization of nanofiber-based acoustic materials. The proposed multiscale modeling approach enables accurate prediction of acoustic behavior from microstructural features, which can be applied to accelerate material design in multiple noise control fields. In architectural acoustics, the approach allows rapid and precise design of broadband, high-efficiency absorbers under low-thickness conditions. Furthermore, the model offers valuable insights for the development of nanofiber composites used in HVAC systems and industrial noise control applications.

Future research will focus on refining the proposed multiscale modeling framework and extending its applicability. First, the optimization of fiber network architecture through parametric and topology-based design could further improve the balance between mechanical strength and acoustic performance. Second, incorporating alternative nanofiber materials—such as carbon, silica, and polymer–inorganic hybrids—may enhance broadband absorption and thermal stability. Moreover, large-scale experimental validation under realistic boundary conditions and complex geometries is necessary to confirm the predictive capability of the model. Finally, integrating the present approach with data-driven or machine-learning-based optimization frameworks could enable intelligent material design, providing rapid prediction and performance tuning of next-generation acoustic materials.

## Figures and Tables

**Table 1:** Summary of bulk density (*ρ*) and measured porosity (*ϕ_m_*) of SP 1 and SP 2 nanofiber samples.

Sample	Bulk Density *ρ* (kg/m^3^)	Measured Porosity *ϕ_m_*
SP 1	47.8	0.956
SP 2	11.2	0.987

**Table 2:** Microstructure parameters of nanofibers.

		Porosity *ϕ*	Flow Resistivity *σ* (Ns/m^4^)	Tortuosity *α_∞_*	Vicious Characteristic Length Λ (µm)	Thermal Characteristic Length Λ’ (µm)
**SBC**	SP 1	0.96	1.22 × 10^6^	1.05	3.64	8.24
	SP 2	0.98	1.56 × 10^6^	1.10	3.32	9.81
NSBC	SP 1	0.96	1.38 × 10^6^	1.05	3.64	8.24
	SP 2	0.98	1.78 × 10^6^	1.10	3.32	9.81

**Table 3:** A comparative summary of the computed microstructural parameters (*ϕ*, *σ*, *α*_∞_, Λ, Λ′) and representative data reported in previous studies for nanofibrous porous materials.

		Porosity *ϕ*	Flow Resistivity *σ* (Ns/m^4^)	Tortuosity α_∞_	Vicious Characteristic Length Λ (µm)	Thermal Characteristic Length Λ’ (µm)
Akasaka et al. [5]	SF 1	0.972	1.35 × 10^6^	2.25	2	7.8
	SF 2	0.969	1.02 × 10^6^	2	4.6	9.1
	SF 3	0.973	7.65 × 10^5^	1.5	10.4	21.5
Sakamoto et al. [32]	SF 1	0.78	5.26 × 10^6^	1.1	4.8	5.5
Authors	SP 1	0.96	1.22 × 10^6^	1.05	3.64	8.24
	SP 2	0.98	1.56 × 10^6^	1.10	3.32	9.81

**Table 4:** Average normal-incidence absorption coefficient ⟨α⟩ (500–6400 Hz) of SP 1 and SP 2 obtained experimentally and from different models.

Sample	Thickness (mm)	Experimental	D–B–M	JCA	Limp Frame Model
SP 1	3	0.376	0.283	0.222	0.379
	5	0.596	0.435	0.362	0.583
	10	0.748	0.615	0.528	0.760
	20	0.815	0.796	0.746	0.834
SP 2	3	0.447	0.293	0.235	0.461
	5	0.716	0.573	0.533	0.698
	10	0.775	0.628	0.554	0.787
	20	0.871	0.815	0.765	0.857

## Data Availability

The original contributions presented in this study are included in the article. Further inquiries can be directed to the corresponding authors.

## Supplementary Materials

The following supporting information can be downloaded at https://www.mdpi.com/article/10.3390/nano15221696/s1, Figure S1: Schematic diagram of the impedance tube showing the incident and reflected sound waves. The distance x_1_ represents the spacing between the sample surface and the nearest microphone.; Table S1: Specifications of the impedance tube measurement system.

## Abbreviation

List of Symbols and Parameters:

Symbol	Unit	Physical Meaning
*μ*	Pa·s	Dynamic viscosity of air
*L*_s_	nm	Slip length
*σ*_v_	—	Momentum accommodation coefficient
Kn	—	Knudsen number
*λ*	nm	Gas mean free path
*d_f_*	nm	fiber diameter
Δ*p*	Pa	Pressure drop across the sample
***u***	m/s	periodic velocity
*h*	m	Sample thickness
*σ*	Ns/m^4^	Flow resistivity; resistance to airflow through porous media
*α_∞_*	—	Tortuosity; ratio of effective to straight acoustic path length
*ϕ*	—	Porosity; fraction of void volume in the total volume
Λ	μm	Viscous characteristic length; controls viscous dissipation scale
Λ′	μm	Thermal characteristic length; controls heat exchange scale
*V_p_*	M^3^	Pore volume
*S_p_*	M^2^	Effective surface area of pores
*ρ*_f_	kg/m^3^	Air density
c_0_	m/s	Speed of sound in air
*Z*_c_	Pa·s/m	Characteristic acoustic impedance
*k*	m^−1^	Complex wave number
ρ(ω)	kg/m^3^	Effective density
*K*(ω)	Pa	Effective bulk modulus
*γ*	—	Ratio of specific heats of air
*p*_A_	Pa	Standard atmospheric pressure
ω	rad/s	Angular frequency
*f*	Hz	Acoustic frequency
Pr	—	Prandtl number
*ρ*	kg/m^3^	Bulk density of the material
*P*_in_	Pa	Inlet pressures of the nanofiber
*P*_out_	Pa	Outlet pressures of the nanofiber
*c*	Pa	Constant pressure drop
*ϕ**_m_*	—	Measured porosity
*ρ_fiber_*	kg/m^3^	Intrinsic density of the nanofiber
*V_t_*	m^3^	Total volume of the domain
