# Supplementary material for: Slip Boundary-Enabled Multiscale Modeling for Sound Absorption Coefficient of Nanofiber Porous Media with High Fidelity
Source: Nanomaterials (Basel). 2025 Nov 9;15(22):1696. doi: 10.3390/nano15221696 (PMC12655751; doi:10.3390/nano15221696)
Supplement: Supplementary file 1 [file nanomaterials-15-01696-s001.zip › nanomaterials-3964921-supplementary.pdf]

Supporting Information

Slip Boundary-Enabled Multiscale Modeling for Sound Absorption Coefficient of Nanofiber Porous Media with High Fidelity

Jiangming Jin[1], *Bohan Cao*[1], *Jietao Huang*[2], *Liyang Jiang*[1], *Ziyi Liu*[2], *Tairong Kuang*[2], *Wei Wu*[3, 4], *Feng Chen*[2], *Yanpei Fei*[2*]

[1] College of Mechanical Engineering, Zhejiang University of Technology, Hangzhou 310014, China

[2] Zhejiang Key Laboratory of Advanced Polymer Materials Modification and Application Technology, College of Material Science and Engineering, Zhejiang University of Technology, Hangzhou 310014, China

[3] ZHANBOQIANYAN New Material Technology Co., Ltd. ®, Jiaxing 314205, China

[4] Zhejiang Joysun Advanced Material Co., Ltd., Jiaxing 314205, China

*Correspondence: feiyanpei@zjut.edu.cn (Y. Fei).

1. SAC measured by the B&K Impendence tube

The sound absorption performance of nanofiber materials under normal incidence was evaluated using an impedance tube following the transfer function method (ISO 10534-2). The measurement system consisted of a Brüel & Kjær (B&K) Type 4206 impedance tube (Denmark) equipped with two 1/4-inch microphones (Type 4187, B&K) and a power amplifier (Type 2716C) to drive the loudspeaker source. The data acquisition and analysis were performed using a dual-channel FFT analyzer (B&K PULSE Type 3560) and PULSE LabShop software (Version 21.0, B&K). The tube has an inner diameter of 29 mm, covering a frequency range of 500–6400 Hz. Nanofiber samples were precisely cut into 29 mm diameter disks using a circular blade to ensure geometric consistency with the tube cross-section.

Two microphones, positioned at a calibrated spacing s_s (20 mm, representing the center-to-center distance between the two microphone ports, as shown in Figure S1), measured sound pressures to derive the complex transfer function H_{12}, defined as the cross-spectrum S_{12} normalized by the auto-spectrum S_{11}. System calibration with a rigid termination ensured phase and amplitude accuracy. The reflection factor r was calculated using:

$$r = \frac{H_{12} - H_I}{H_R - H_{12}} e^{j2k_0 x_1} \quad (1)$$

where H_I and H_R represent the incident and reflected wave transfer functions, k_0 is the complex wavenumber, and x_1 denotes the distance from the sample surface to the nearest microphone(70mm). The normal incidence absorption coefficient α was then determined as:

$$\alpha = 1 - |r|^2 \quad (2)$$

To facilitate reproducibility and comparison, the key specifications of the measurement system are summarized in Table S1.

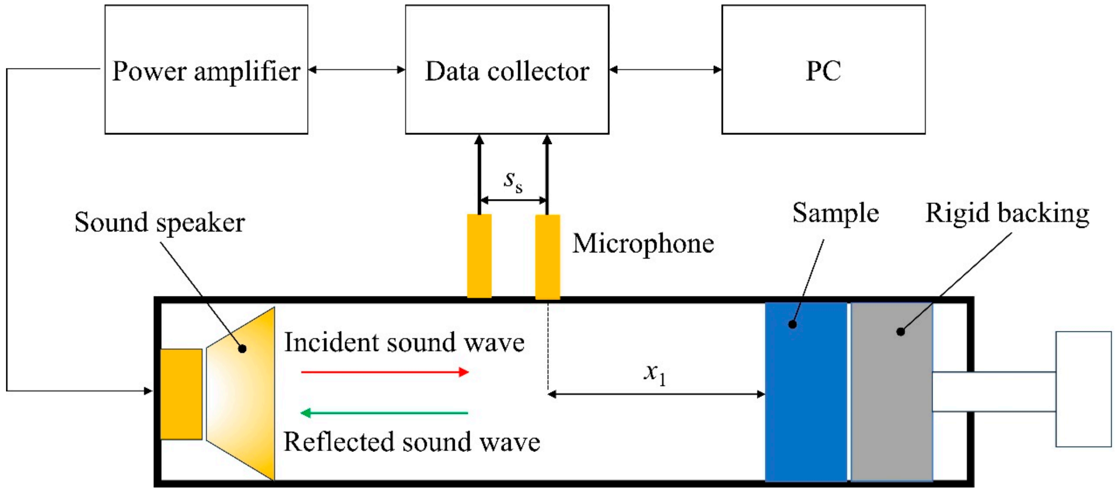

Figure. S1. Schematic diagram of the impedance tube showing the incident and reflected sound waves. The distance x_1 represents the spacing between the sample surface and the nearest microphone.

Table S1. Specifications of the impedance tube measurement system

Component	Model / Manufacturer	Specification / Function
**Impedance Tube**	B&K Type 4206	Frequency range: 500–6400 Hz; Inner diameter: 29 mm
**Microphones**	B&K Type 4187 (¼ inch)	Sensitivity: 50 mV/Pa; Frequency range: 20–20,000 Hz
**Power Amplifier**	B&K Type 2716C	Drives loudspeaker source
**Data Acquisition System**	B&K PULSE Type 3560	Two-channel FFT analyzer
**Software**	B&K PULSE LabShop (v21.0)	Transfer function and SAC calculation
**Calibration Method**	Two-microphone transfer function	Conforms to ISO 10534-2
